# Anticholinergic burden measures, symptoms, and fall-associated risk in older adults with polypharmacy: Development and validation of a prognostic model

**DOI:** 10.1371/journal.pone.0280907

**Published:** 2023-01-23

**Authors:** Truc Sophia Dinh, Andreas D. Meid, Henrik Rudolf, Maria-Sophie Brueckle, Ana I. González-González, Veronika Bencheva, Matthias Gogolin, Kym I. E. Snell, Petra J. M. Elders, Petra A. Thuermann, Norbert Donner-Banzhoff, Jeanet W. Blom, Marjan van den Akker, Ferdinand M. Gerlach, Sebastian Harder, Ulrich Thiem, Paul P. Glasziou, Walter E. Haefeli, Christiane Muth

**Affiliations:** 1 Institute of General Practice, Goethe University Frankfurt, Frankfurt am Main, Germany; 2 Department of Clinical Pharmacology and Pharmacoepidemiology, Heidelberg University Hospital, Heidelberg, Germany; 3 Department of Medical Informatics, Biometry and Epidemiology, Ruhr-University Bochum, Bochum, Germany; 4 HELIOS University Clinic Wuppertal, Philipp Klee-Institute for Clinical Pharmacology, University of Witten / Herdecke, Witten, Germany; 5 Centre for Prognosis Research, School of Medicine, Keele University, Staffordshire, United Kingdom; 6 Amsterdam UMC, General Practice and Elderly Care Medicine, Amsterdam Public Health Research Institute, Vrije Universiteit Amsterdam, Amsterdam, The Netherlands; 7 Department of General Practice / Family Medicine, Philipps University Marburg, Marburg, Germany; 8 Department of Public Health and Primary Care, Leiden University Medical Center, Leiden, The Netherlands; 9 Department of Family Medicine, Care and Public Health Research Institute, Maastricht University, Maastricht, The Netherlands; 10 Department of Public Health and Primary Care, Academic Centre of General Practice, KU Leuven, Leuven, Belgium; 11 Institute of Clinical Pharmacology, Goethe University Frankfurt, Frankfurt, Germany; 12 Department of Geriatrics, Immanuel Albertinen Diakonie, Albertinen-Haus, Hamburg, Germany; 13 University Clinic Eppendorf, Hamburg, Germany; 14 Faculty of Health Sciences and Medicine, Bond University, Robina, QLD, Australia; 15 Department of General Practice and Family Medicine, Medical Faculty East-Westphalia, University of Bielefeld, Bielefeld, Germany; Clinca Geriatrica, ITALY

## Abstract

**Background:**

Anticholinergic burden has been associated with adverse outcomes such as falls. To date, no gold standard measure has been identified to assess anticholinergic burden, and no conclusion has been drawn on which of the different measure algorithms best predicts falls in older patients from general practice. This study compared the ability of five measures of anticholinergic burden to predict falls. To account for patients’ individual susceptibility to medications, the added predictive value of typical anticholinergic symptoms was further quantified in this context.

**Methods and findings:**

To predict falls, models were developed and validated based on logistic regression models created using data from two German cluster-randomized controlled trials. The outcome was defined as “≥ 1 fall” vs. “no fall” within a 6-month follow-up period. Data from the RIME study (*n* = 1,197) were used in model development, and from PRIMUM (*n* = 502) for external validation. The models were developed step-wise in order to quantify the predictive ability of anticholinergic burden measures, and anticholinergic symptoms. In the development set, 1,015 patients had complete data and 188 (18.5%) experienced ≥ 1 fall within the 6-month follow-up period. The overall predictive value of the five anticholinergic measures was limited, with neither the employed anticholinergic variable (binary / count / burden), nor dose-dependent or dose-independent measures differing significantly in their ability to predict falls. The highest *c*-statistic was obtained using the German Anticholinergic Burden Score (0.73), whereby the optimism-corrected *c*-statistic was 0.71 after interval validation using bootstrapping and 0.63 in the external validation. Previous falls and dizziness / vertigo had the strongest prognostic value in all models.

**Conclusions:**

The ability of anticholinergic burden measures to predict falls does not appear to differ significantly, and the added value they contribute to risk classification in fall-prediction models is limited. Previous falls and dizziness / vertigo contributed most to model performance.

## Introduction

Drugs with anticholinergic (ACh) properties are the most frequently prescribed potentially inappropriate medications in older adults [1], irrespective of whether the ACh effects are desired (as with spasmolytics), or are merely a side effect (as with some antipsychotics). They have been associated with a variety of adverse drug reactions (e.g., dry mouth, blurred vision, and drowsiness) and adverse outcomes, most notably including delirium, cognitive decline and falls [2–6]. Falling, in particular, is one of the main causes of disability, injuries and death in older patients, and is associated with hospital admissions, reduced health-related quality of life and increased health care costs [7–9].

Over the past 20 years, more than 20 measures have been developed to quantify ACh burden and help clinicians reduce their adverse effects. No gold standard measure yet exists, presumably because the employed metrics differ considerably in terms of, for example, the (number of) included drugs and the way (cumulative) ACh burden is calculated [3, 5, 10–14]. For instance, the same drug (e.g., furosemide) can be rated as having low or high activity, depending on the measure’s definition [15]. While most measures use a scoring system from 0 (none) to 3 (high) to describe pharmacological interactions with muscarinic receptors, some exceptions [16–19] also consider drug dosage. Many studies have examined the ability of these ACh burden measures to predict adverse outcomes, including falls [3, 5, 10–14].

However, the association between specific measures of ACh burden and falls has been inconsistently described in the literature [3, 12, 20, 21]. While the Drug Burden Index (DBI) [16] and the Anticholinergic Risk Scale (ARS) [22] have shown some degree of association with falls [3, 12, 21, 22], it has not yet been possible to draw a general conclusion on which ACh measure best predicts falls in older patients [21]. Older patients, in particular, are at increased risk of the accumulating consequences of multimorbidity and subsequent polypharmacy [16, 23–26]. In addition, unspecific ACh effects such as dizziness and blurred vision may be overlooked by clinicians and mistakenly interpreted as age-related symptoms [27]. Even though these symptoms may not directly cause substantial harm, they can indirectly lead to falls and other adverse outcomes [28]. It remains unclear which, if any, existing ACh measures can help predict falls in clinical practice, whereby it is also important that ACh symptoms are taken into account.

The aim of this study was therefore to compare the ability of five ACh burden measures to predict falls (and, if possible, to identify the one that performs best), and thereby to quantify the added predictive value of ACh symptoms.

## Methods

The rationale behind and methodology used in this study have been described in detail in a study protocol [29]. We therefore provide only a brief summary here, in which we also describe necessary adaptions (a summary of adaptions to the study protocol is included in the S1 Text).

### Source of data

Data from the binational PROPERmed database were used in model development and validation. In PROPERmed, individual participant data from five German and Dutch cluster-randomized controlled trials (cRCTs) were combined for pooled modelling purposes [30–32]. For this study, variables on ACh symptoms, a history of falls, and cognitive function were additionally taken from the studies. The RIME study (Reduction of potentially Inadequate Medication in the Elderly [33, 34]) and the PRIMUM study (PRIoritizing Multimedication in Multimorbidity [35]) were the only two cRCTs from PROPERmed to include this data and were therefore considered in this analysis. Both trials aimed to optimize medication in older German general practice (GP) patients. Data from the RIME trial was chosen for model development due to its larger sample size and higher number of events, and data from PRIMUM was used for external validation.

### Participants

RIME included 1,197 older patients from 139 GP practices, and PRIMUM 502 patients from 72 GP practices. In RIME, patients were included if they were ≥ 70 years old and were prescribed ≥ 6 chronic medications. PRIMUM included patients aged ≥ 60 years, with ≥ 3 chronic conditions and prescriptions for ≥ 5 chronic medications. Patients with dementia, cognitive impairment, or a reduced life expectancy (RIME: ≤ 6 months, PRIMUM: ≤ 12 months), were excluded from participation.

### Outcome

The study outcome was a binary indicator defined as “≥ 1 fall(s)” vs. “no fall(s)” within a 6-month follow-up period. Information on falls was self-reported and collected during patient interviews.

### Predictors

For this study, candidate predictors were pre-selected based on a literature review, predictor availability, and clinical reasoning [29]. Candidate predictors were collected at baseline and included variables associated with:

- Sociodemographics and lifestyle (age, sex, living situation, educational level [36], and smoking status);
- Morbidity (in accordance with a list of 24 chronic conditions [30], number of chronic conditions);
- Health-status and well-being (pain, health-related quality of life [37], functional status [38], cognitive function, all-cause hospital admissions, and history of falls);
- Medication (number of drugs and variables to characterize ACh medications (see below));
- ACh symptoms (list of symptoms).

ACh-related symptoms were measured as binary indicators in both trials. The development and validation datasets shared three ACh symptoms (dizziness / vertigo, problems urinating, and stomach pain), while four further ACh symptoms were only present in the development dataset (drowsiness / fatigue, dry mouth, itching, and constipation), and one in the validation dataset (palpitations). In both trials, the history of falls was documented for 6-months before baseline. In RIME, cognitive function was measured using a word list that participants were asked to reproduce, while in PRIMUM, a verbal fluency test was employed [39]. To harmonize the different cognitive assessments in the two studies, quartiles were calculated and cognitive impairment was interpreted as no / mild / medium / severe.

Anticholinergic burden was calculated based on ATC codes and according to five different scales / equations:

1. Anticholinergic Risk Scale (ARS) [22];
2. Anticholinergic Drug Scale (ADS) [40];
3. German Anticholinergic Burden Score (GerABS) [41];
4. Muscarinic Acetylcholinergic Receptor ANTagonist Exposure scale (MARANTE) [17];
5. German Drug Burden Index (GerDBI).

Of these five ACh measures, three used scores ranging from 0 to 3 [22, 40, 41], and two used equations that took drug dosage into account ([17] and the GerDBI). Details on the GerDBI, which was recently developed as part of the COFRAIL-study [42] and is based on the Drug Burden Index by Hilmer et al. [16, 43], will be published elsewhere. The scales were developed in Germany ([41] and GerDBI), the United States [22, 40], and Belgium [17]. In developing the model, three variables associated with ACh burden were calculated for each of the included scales / equations: a binary variable (prescription of ≥ 1 ACh medication(s)), a “count” variable (number of ACh medications per patient), and a “burden” variable (cumulative ACh burden / load for all of a patient’s medications).

### Statistical analysis

#### Model development and performance

Logistic regression analysis was used to develop the model, whereby our intention was to quantify the predictive ability of ACh burden measures, and ACh symptoms. We therefore developed the prognostic model stepwise (see below). We used backwards selection based on Akaike’s information criterion (AIC) and determined the suitable functional form for continuous variables using the multivariable fractional polynomial (MFP) approach [44, 45]. Variables (age and sex) that we considered clinically relevant were forced back into the model. The model was developed as follows:

In step 1, a base model (Model 1) was built to predict falls within a 6-month follow-up period using variables associated with sociodemographic / lifestyle, morbidity, health-status / well-being, and number of drugs.

In step 2, the aforementioned variables associated with ACh drug burden use (binary indicator / total (ACh drug) count / cumulative ACh burden) were separately added to the base model, bringing the number of different models to 15 (Models 2.1–2.15). In addition to age and sex, the ACh variable of interest was also considered mandatory in the corresponding models.

In step 3, ACh symptom variables were added to the 15 models developed in step 2 to quantify their additional predictive value. As some symptoms were not available in both development and validation datasets, two different types of model were developed at this stage:

- Step 3A: models that considered only shared symptoms (Models 3.1–3.15), and
- Step 3B: models that included all symptoms from the development dataset (Models 3.16–3.30).

An additional Model 4 (base model + ACh symptoms) was developed in step 4, with the aim to determine the added predictive value from ACh symptoms separately. Step 3B models and an additional Model 4 were only developed for exploratory purposes.

After developing the models, the best-performing step 3A model was selected for internal and external validation (based on the highest *c*-statistic and lowest AIC) [44]. The *c*-statistic and the model fit using AIC (in-sample) were used to assess the performance of models and to discriminate between them [44]. Added predictive value was quantified by comparing differences in area under the curve (AUC) and using integrated discrimination improvement (IDI) [46].

#### Internal validation

The selected model was internally validated using bootstrapping. This involved creating a bootstrap sample and using it to develop the model and determine the predictive performance in both the bootstrap and the original sample. These steps were repeated 100 times and optimism was estimated. Mean optimism was then subtracted from the apparent performance of the original model to obtain optimization-adjusted performance estimates [47]. To adjust for overfitting, we determined the uniform shrinkage factor by shrinking the regression coefficients. The performance and discriminative ability of the models were compared before and after internal validation.

#### External validation

The selected 3A model was externally validated using data from the PRIMUM study. Discriminative ability was assessed using *c*-statistic, and calibration of actual and predictive risk by estimating the expected / observed ratio. In addition, the calibration slope and calibration-in-the-large were calculated and a calibration plot was produced [48].

### Sample size

We used the *pmsampsize* package in R to calculate the minimum sample size [49, 50]. Due to the nature of the study, this calculation was performed retrospectively and had only exploratory character. Based on the number of candidate predictors, empirical *c*-statistics (0.87) [51] and the given prevalence in the complete-case population (18.5%), the minimum sample size required to minimize the model’s potential for overfitting was calculated to be *n* = 1,131 with 210 events. We consider this acceptable with view of the size of the complete-case population of the development cohort (*n* = 1,015 with 188 events).

### Missing data

Multiple imputation techniques were used to handle missing data [47, 52]. The nine imputed datasets corresponded to the percentage of incomplete observations [53, 54], which was approximately 8.5% in the development dataset. The selected model was developed for each of the nine multiply imputed datasets, and pooled estimates were obtained and compared with the results of the complete-case analysis [55].

### Technical information and reporting

Statistical analyses were conducted using R version 4.1.2 (R Foundation for Statistical Computing, Vienna, Austria). The present manuscript follows the Transparent Reporting of a multivariable model for Individual Prognosis or Diagnosis (TRIPOD) statement [56].

## Results

### Participants

The complete-case populations of RIME and PRIMUM included 1,015 and 348 patients respectively. In RIME patients, mean age was 77 years, 50% were female, and 92% had a low or medium level of education. In PRIMUM participants, mean age was 72 years, 55% were female, and 90% had a low or medium level of education. The average number of chronic conditions was six in RIME and five in PRIMUM patients. RIME participants were taking an average of nine chronic medications and had two ACh symptoms, while PRIMUM patients used eight medications and reported one ACh symptom. In RIME, between 12.3% and 78.0% of patients used ≥ 1 ACh medication depending on which scale was used (ARS 12.3%, MARANTE 19.5%, ADS 37.9%, GerDBI 52.3%, and GerABS 78.0%). Anticholinergic drug use in PRIMUM varied between 8.3% and 72.7% (ARS 8.3%, MARANTE 17.8%, ADS 30.2%, GerDBI 39.1%, and GerABS 72.7%). The S1 Table provides a detailed overview of the characteristics of the complete-case population including the percentage of missing values, odds ratios, and confidence intervals from unadjusted bivariate analyses.

### Model development

Of the complete-case population of the development set, 188 (18.5%) patients experienced at least one fall within the 6-month follow-up period. Table 1 shows overall prognostic variables stratified per observed outcome for both the development and validation sets. Bivariate analyses from the development set showed that patients that fell tended to have a history of falls (OR = 5.9), to suffer from pain (OR = 2.8) or dizziness (OR = 2.3), to have a higher number of ACh symptoms (OR = 1.2), and to have reduced functional status (OR = 1.2) (see S1 Table). Other predictors that were significantly associated with falls were, for example, the number of chronic conditions, number of chronic medications, health-related quality of life, female sex, and a previous admission to hospital. Anticholinergic drug use was higher in fallers measured with all five ACh scales / equations. Apart from the ARS, bivariate analyses showed an association between falls and both ACh “count” and “burden” variables from all ACh measures, while, except for MARANTE, no binary ACh variables were associated with falls.

**Table 1:** Baseline characteristics of development and validation sets stratified per observed outcome (complete-case populations).

Type	Variables	Categories / measurement unit	Development set (*n* = 1,015)		Validation set (*n* = 348)	
			No fall (*n* = 827)	Fall (*n* = 188)	No fall (*n* = 293)	Fall (*n* = 55)
Sociodemographic- and lifestyle-related	Intervention status, no. (%)	Control	424 (51.3)	81 (43.1)	147 (50.2)	29 (52.7)
		Intervention	403 (48.7)	107 (56.9)	146 (49.8)	26 (47.3)
	Age, mean (SD)	Years	76.7 (4.8)	77.1 (4.8)	72.2 (7.1)	72.9 (6.6)
	Sex, no. (%)	Male	432 (52.2)	76 (40.4)	133 (45.4)	25 (45.5)
		Female	395 (47.8)	112 (59.6)	160 (54.6)	30 (54.5)
	Living situation, no. (%)	Living at home	798 (96.5)	179 (95.2)	292 (99.7)	55 (100)
		Institutionalized living	29 (3.5)	9 (4.8)	1 (0.3)	0 (0)
	Educational level, no. (%)	Low	147 (17.8)	36 (19.1)	182 (62.1)	29 (52.7)
		Medium	607 (73.4)	140 (74.5)	83 (28.3)	19 (34.5)
		High	73 (8.8)	12 (6.4)	28 (9.6)	7 (12.7)
	Smoking, no. (%)	Smoker	57 (6.9)	5 (2.7)	28 (9.6)	2 (3.6)
		Ex-Smoker	397 (48.0)	89 (47.3)	119 (40.6)	25 (45.5)
		Non-Smoker	373 (45.1)	94 (50.0)	146 (49.8)	28 (50.9)
Morbidity-related	Hypertension, no. (%)	Yes	726 (87.8)	173 (92.0)	247 (84.3)	45 (81.8)
	Diabetes mellitus, no. (%)	Yes	362 (43.8)	92 (48.9)	146 (49.8)	28 (50.9)
	Coronary heart disease, no. (%)	Yes	353 (42.7)	82 (43.6)	132 (45.1)	27 (49.1)
	Osteoarthritis, no. (%)	Yes	359 (43.4)	98 (52.1)	165 (56.3)	25 (45.5)
	COPD / asthma, no. (%)	Yes	190 (23.0)	36 (19.1)	81 (27.6)	15 (27.3)
	Vision problems, no. (%)	Yes	375 (45.3)	100 (53.2)	51 (17.4)	9 (16.4)
	Hearing problems, no. (%)	Yes	315 (38.1)	88 (46.8)	8 (2.7)	1 (1.8)
	Cancer, no. (%)	Yes	153 (18.5)	47 (25.0)	44 (15.0)	9 (16.4)
	Heart failure, no. (%)	Yes	275 (33.3)	59 (31.4)	45 (15.4)	11 (20)
	Cerebrovascular disease, no. (%)	Yes	109 (13.2)	28 (14.9)	59 (20.1)	9 (16.4)
	Osteoporosis, no. (%)	Yes	164 (19.8)	48 (25.5)	36 (12.3)	4 (7.3)
	Depression, no. (%)	Yes	92 (11.1)	29 (15.4)	51 (17.4)	5 (9.1)
	Rheumatoid / seropositive arthritis, no. (%)	Yes	158 (19.1%)	38 (20.2%)	18 (6.1%)	3 (5.5%)
Morbidity- related (cont.)	Atherosclerosis / peripheral vascular disease, no. (%)	Yes	241 (29.1)	67 (35.6)	50 (17.1)	9 (16.4)
	Parkinsonism, no. (%)	Yes	17 (2.1)	4 (2.1)	4 (1.4)	1 (1.8)
	HIV / AIDS, no. (%)	Yes	0 (0)	0 (0)	0 (0)	0 (0)
	Lipid disorder, no. (%)	Yes	485 (58.6)	108 (57.4)	111 (37.9)	28 (50.9)
	Hyperuricemia / gout, no. (%)	Yes	249 (30.1)	55 (29.3)	8 (2.7)	3 (5.5)
	Thyroid disorders, no. (%)	Yes	247 (29.9)	71 (37.8)	56 (19.1)	13 (23.6)
	Gastric or duodenal ulcer, no. (%)	Yes	93 (11.2)	26 (13.8)	34 (11.6)	2 (3.6)
	Liver disorder, no. (%)	Yes	48 (5.8)	12 (6.4)	34 (11.6)	5 (9.1)
	Urinary disease, no. (%)	Yes	152 (18.4)	43 (22.9)	89 (30.4)	15 (27.3)
	Anemia, no. (%)	Yes	75 (9.1)	28 (14.9)	12 (4.1)	2 (3.6)
	No. of chronic conditions, mean (SD)		6.3 (2.5)	7.1 (2.7)	5.0 (2.0)	4.9 (1.5)
Health-status and well-being related	Pain, no. (%)	Yes	579 (70.0)	163 (86.7)	256 (87.4)	49 (89.1)
	Quality of life, mean (SD)	Score	0.7 (0.2)	0.6 (0.2)	0.7 (0.2)	0.7 (0.3)
	Functional status, mean (SD)	Score	2.5 (2.4)	3.7 (2.6)	2.5 (2.7)	3.7 (3.0)
	Cognitive impairment, no. (%)	Severe	362 (43.8)	80 (42.6)	126 (43)	31 (56.4)
		Medium	188 (22.7)	44 (23.4)	70 (23.9)	12 (21.8)
		Mild	128 (15.5)	22 (11.7)	49 (16.7)	7 (12.7)
		No	149 (18.0)	42 (22.3)	48 (16.4)	5 (9.1)
	All-cause hospital admissions, no. (%)	Yes	322 (38.9)	54 (28.7)	46 (15.7)	12 (21.8)
	History of falls at baseline, no. (%)	0–1 fall	804 (97.2)	161 (85.6)	287 (98.0)	43 (78.2)
		≥ 2 falls	23 (2.8)	27 (14.4)	6 (2.0)	12 (21.8)
Medication-related	No. of drugs, mean (SD)		8.4 (2.5)	9.2 (2.9)	7.8 (2.6)	8.5 (2.8)
	ARS binary, no. (%)	Yes	98 (11.9)	27 (14.4)	20 (6.8)	9 (16.4)
	ARS count, mean (SD)		0.1 (0.4)	0.2 (0.5)	0.1 (0.3)	0.2 (0.4)
	ARS burden, mean (SD)	Score	0.2 (0.7)	0.3 (0.9)	0.2 (0.8)	0.4 (1.2)
	ADS binary, no. (%)	Yes	307 (37.1)	78 (41.5)	89 (30.4)	16 (29.1)
Medication-related (cont.)	ADS count, mean (SD)		0.6 (1.0)	0.6 (0.8)	0.4 (0.7)	0.4 (0.8)
	ADS burden, mean (SD)	Score	0.5 (0.7)	0.9 (1.3)	0.7 (1.3)	0.8 (1.8)
	GerABS binary, no. (%)	Yes	644 (77.9)	148 (78.7)	210 (71.7)	43 (78.2)
	GerABS count, mean (SD)		1.3 (1.0)	1.5 (1.3)	1.1 (1.0)	1.5 (1.2)
	GerABS burden, mean (SD)	Score	1.5 (1.4)	1.9 (1.8)	1.6 (1.8)	1.9 (1.7)
	MARANTE binary, no. (%)	Yes	153 (18.5)	48 (25.5)	46 (15.7)	16 (29.1)
	MARANTE count, mean (SD)		0.22 (0.5)	0.3 (0.6)	0.2 (0.5)	0.4 (0.7)
	MARANTE burden, mean (SD)	Score	0.3 (0.9)	0.6 (1.2)	0.3 (1.2)	0.4 (0.7)
	GerDBI binary, no. (%)	Yes	393 (47.5)	97 (51.6)	111 (37.9)	25 (45.5)
	GerDBI count, mean (SD)		0.84 (1.1)	1.1 (1.3)	0.6 (0.9)	0.9 (1.4)
	GerDBI burden, mean (SD)	Score	0.5 (0.6)	0.6 (0.8)	0.3 (0.5)	0.5 (0.8)
Symptoms	No. of symptoms, mean (SD)		2.1 (1.6)	2.6 (1.6)	0.9 (0.9)	0.9 (0.8)
	Dizziness / vertigo, no. (%)	Yes	306 (37.0)	108 (57.5)	79 (27)	18 (32.7)
	Problems urinating, no. (%)	Yes	152 (18.4)	40 (21.3)	65 (22.2)	10 (18.2)
	Stomach pain, no. (%)	Yes	142 (17.2)	30 (16.0)	57 (19.5)	9 (16.4)
	Drowsiness / fatigue, no. (%)	Yes	328 (39.7)	91 (48.4)	n.a.	n.a.
	Dry mouth, no. (%)	Yes	376 (45.5)	99 (52.7)	n.a.	n.a.
	Itching, no. (%)	Yes	218 (26.4)	62 (33.0)	n.a.	n.a.
	Constipation, no. (%)	Yes	223 (27.0)	66 (35.1)	n.a.	n.a.
	Palpitations, no. (%)	Yes	n.a.	n.a.	52 (17.7)	7 (12.7)

Binary: prescription of ≥ 1 anticholinergic medication; count: number of anticholinergic medications per patient; burden: cumulative anticholinergic burden / load of all of a patient’s medications.

**Abbreviations:** ARS–Anticholinergic Risk Scale [22]; ADS–Anticholinergic Drug Scale [40]; GerABS–German Anticholinergic Burden Score [41]; MARANTE–Muscarinic Acetylcholinergic Receptor ANTagonist Exposure Scale [17]; GerDBI–German Drug Burden Index; n.a.–not applicable

### Model development and internal validation

When the base model for falls was developed using candidate predictors from the categories of sociodemographics / lifestyle, morbidity, health-status / well-being, and number of drugs (step 1), variable selection using MFP and AIC yielded the best-performing Model 1 with a *c*-statistic of 0.712 (see S2 Table). The addition of ACh variables (step 2) brought the number of different models to 15, of which the *c*-statistics ranged from 0.712 to 0.714 (S3 Table). When the 15 models were extended to include ACh symptom variables that were present in both trials (step 3A), they produced *c*-statistics between 0.724 and 0.732 (see Table 2).

**Table 2:** Comparison of model performances from step 3A.

Model	Predictors	*c*-statistic
3.1	Base model + ARS binary + symptoms	0.731
3.2	Base model + ARS count + symptoms	0.730
3.3	Base model + ARS burden + symptoms	0.731
3.4	Base model + ADS binary + symptoms	0.727
3.5	Base model + ADS count + symptoms	0.727
3.6	Base model + ADS burden + symptoms	0.725
3.7	Base model + MARANTE binary + symptoms	0.730
3.8	Base model + MARANTE count + symptoms	0.730
3.9	Base model + MARANTE burden + symptoms	0.730
3.10*	Base model + GerABS binary + symptoms	0.732
3.11	Base model + GerABS count + symptoms	0.724
3.12	Base model + GerABS burden + symptoms	0.730
3.13	Base model + GerDBI binary + symptoms	0.726
3.14	Base model + GerDBI count + symptoms	0.726
3.15	Base model + GerDBI burden + symptoms	0.730

*selected model

Binary: ≥ 1 anticholinergic medications; count: number of anticholinergic medications per patient; burden: cumulative anticholinergic burden / load of all of a patient’s medications.

Abbreviations: ACh–anticholinergic; ARS–Anticholinergic Risk Scale [22]; ADS–Anticholinergic Drug Scale [40]; GerABS–German Anticholinergic Burden Score [41]; MARANTE–Muscarinic Acetylcholinergic Receptor ANTagonist Exposure Scale [17]; GerDBI–German Drug Burden Index.

The best-performing model included the binary variable of the German Anticholinergic Burden Score by Kiesel et al. [41]. Based on the AUC, the discrimination performance of the model was 0.732. Bootstrap resampling resulted in a uniform shrinkage factor of 0.86 (average calibration slope from bootstrap samples). This was applied to the model to adjust for overfitting, and resulted in an optimism-adjusted AUC of 0.705 and optimism-adjusted regression coefficients (see Table 3).

**Table 3:** Selected model for falls within 6-months of follow-up (developed and internally validated).

Intercept and predictors	Units	Original model		Optimism-adjusted model	
		Regression Coefficient	*p*-value	Regression Coefficient	*p*-value
Intercept		-3.51	0.02	-3.12	0.04
History of falls at baseline	≥ 2 falls	1.55	<0.001	1.33	<0.001
Dizziness / vertigo	Yes/No	0.60	0.001	0.51	0.004
COPD / asthma	Yes/No	-0.58	0.009	-0.50	0.03
Pain	Yes/No	0.67	0.007	0.58	0.02
All-cause hospital admissions	Yes/No	-0.49	0.009	-0.42	0.03
Functional status	Score	0.92	0.01	0.08	0.03
Stomach pain	Yes/No	-0.48	0.04	-0.41	0.08
Intervention status	Intervention	0.25	0.16	0.21	0.22
Hearing problems	Yes/No	0.27	0.14	0.23	0.20
Cancer	Yes/No	0.31	0.13	0.27	0.20
No. of drugs	Frequency	0.87	0.008	0.08	0.02
GerABS binary	Yes/No	-0.12	0.56	-0.11	0.62
Sex	Female	0.35	0.05	0.30	0.10
Age	Years	-0.08	0.96	0.00	0.97

Abbreviation: GerABS–German Anticholinergic Burden Score [41].

### External validation

**Fig 1:**
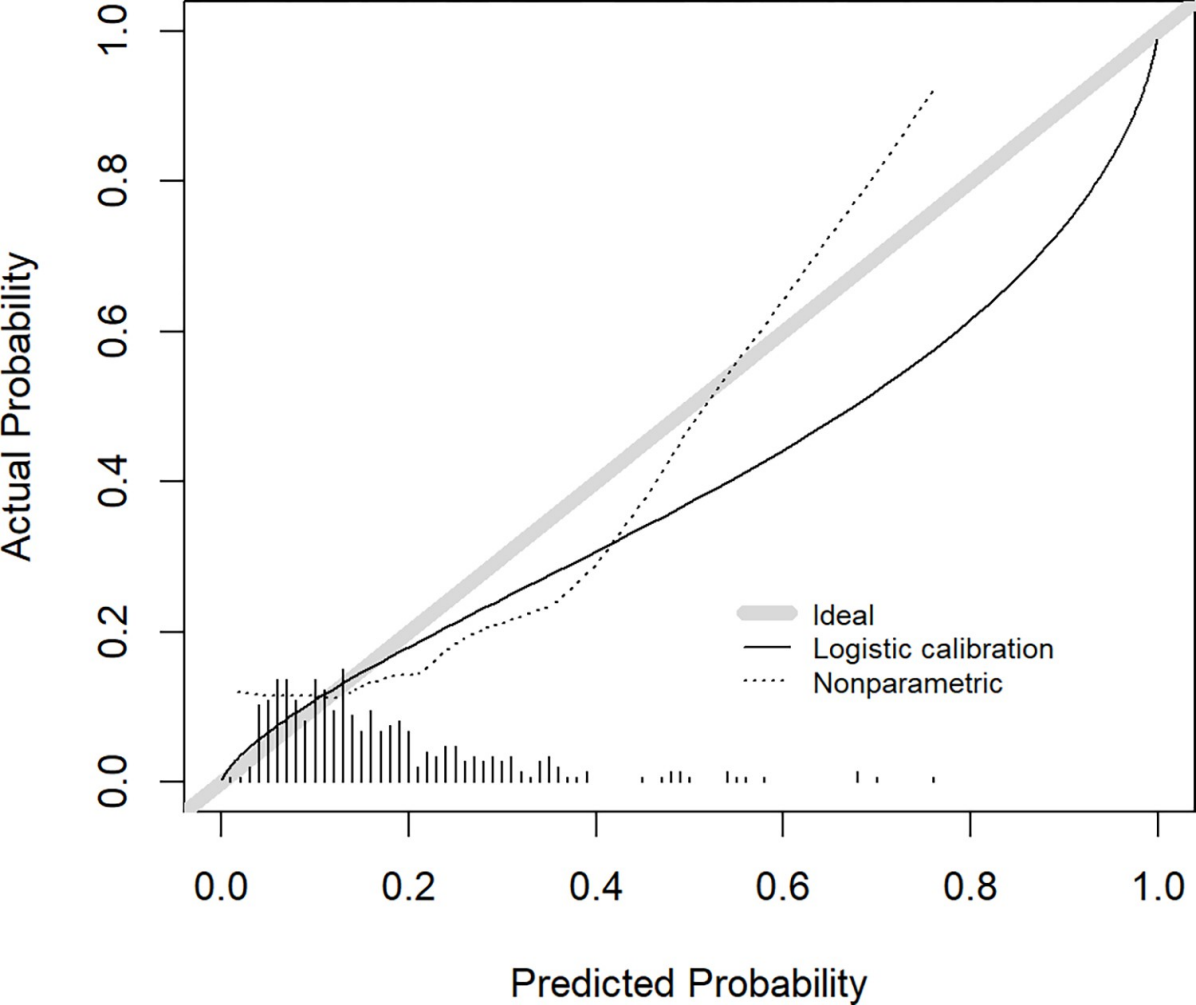
Calibration plot of actual versus the predicted probability in the external validation.

After external validation, *c*-statistic decreased from 0.732 to 0.632. The expected / observed ratio was 1.09, indicating that the model over-predicts the total number of events. This was supported by a calibration slope of 0.716. The average predicted risk was 17.3% while the observed risk was 16.4%, showing that the model over-estimated the incidence by 0.9% (calibration-in-the-large). A calibration plot is shown in Fig 1.

### Additional analyses

**Fig 2:**
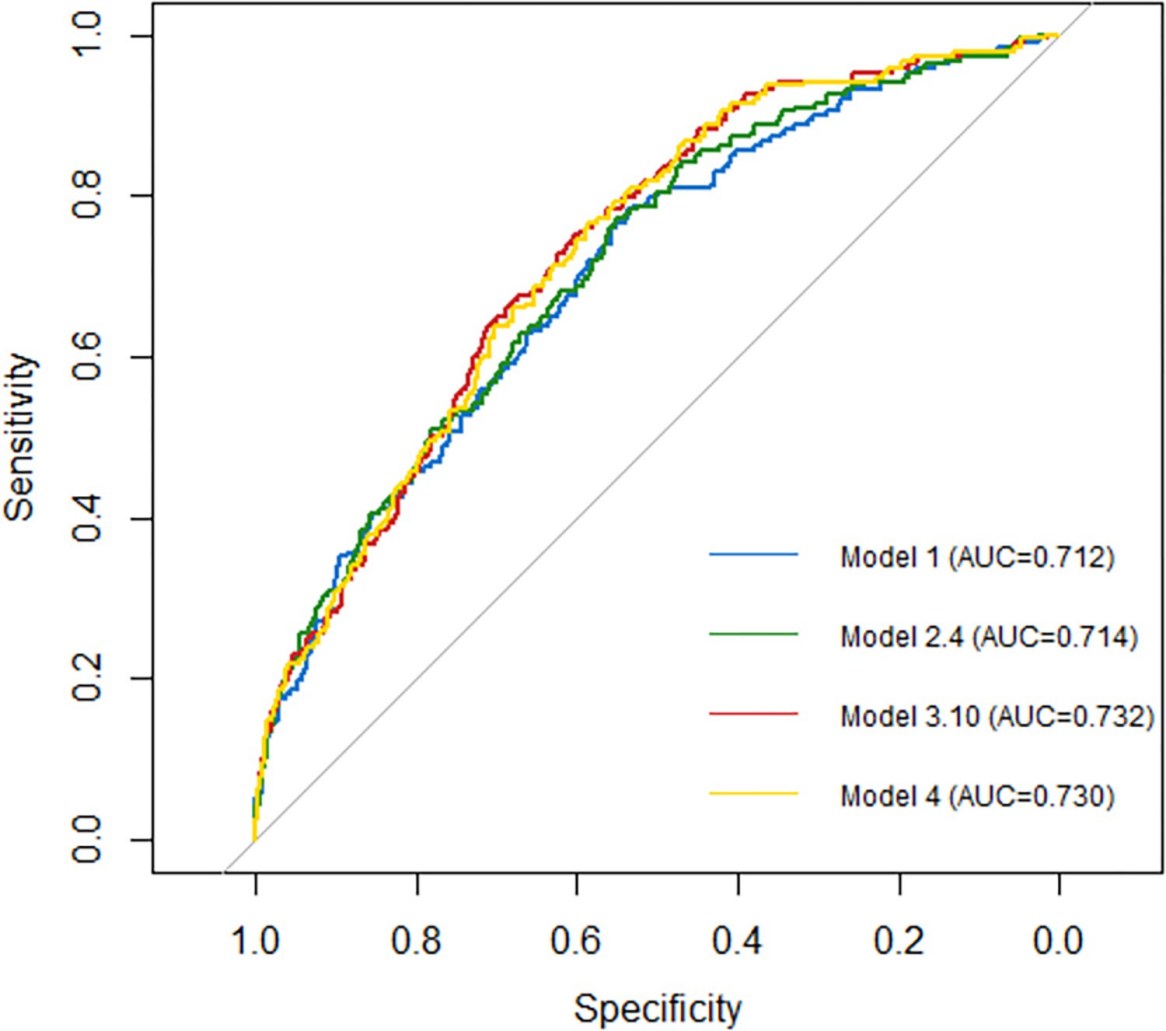
Receiver operator curves of the best-performing models in each model development step (AUC, area under the curve).

After multiple imputation of missing data, pooled estimates did not reveal substantial differences to the results of the complete-case analysis (see S4 Table). Exploratory analyses showed that (1) in terms of the *c*-statistic and AIC, models that included all symptoms (step 3B) did not significantly differ from models based on shared symptoms, and (2) the development of a model without variables on ACh use (Model 4, see S5 Table) resulted in a model of almost identical discriminative ability to the selected model that included them (AUC_w/o ACh variables_ = 0.730 vs. AUC_with ACh variables_ = 0.732). Fig 2 shows the ROC curves of the best-performing models for all steps, and reveals minor differences in the discriminative ability of models with and without ACh variables (Model 3.10 vs. Model 4). An overview of the increase in discrimination in terms of Δ AUC and IDI is presented in Table 4.

**Table 4:** Increase in discrimination.

Model	Type of predictors	AUC	Δ AUC*	IDI* (%)
1	Base model	0.712	-	-
2.4	Base model + Ach	0.714	0.002	0.004 (+0.3%)
3.10	Base model + ACh + symptoms	0.732	0.027	0.019 (+2.8%)
4	Base model + symptoms	0.730	0.025	0.019 (+2.5%)

*Ref. = base model

ACh–anticholinergic; AUC–area under the curve; IDI–integrated discrimination improvement.

Sensitivity analysis explored the effect of the intervention on the model’s outcome. The results of sensitivity analysis, in which intervention status was excluded from model development and validation, indicate that the *c*-statistic decreased from 0.712 to 0.710 for the base model, and from 0.732 to 0.727 for the full model, while the *c*-statistic of the validated model increased from 0.632 to 0.635 (see S5 Table).

## Discussion

Our findings indicate that there are no significant differences in the ability of ACh scales / equations to predict falls. Furthermore, neither the type of ACh variable that was operationalized (i.e., binary indicator / total (ACh drug) count / cumulative ACh burden), nor the use of dose-dependent or dose-independent calculations, improved discrimination. The results indicate that measures of ACh burden hardly improve model performance at all, while measures of ACh symptoms do, at least marginally. In this respect, dizziness / vertigo may be interpreted as a typical side effect of ACh drugs and as such a good predictor of falls. A fundamental distinction must be made between association and prediction. While ACh burden measures may be more or less strongly associated with falls, their added predictive value *beyond* other significant predictors appears to be limited.

The use of ACh medications varied greatly in our study depending on the ACh metric used (12.3%– 78.0%). This result agrees with previous findings reporting that ACh use in a variety of clinical settings and patient populations ranged from 9%– 80% [12, 57–60] and are presumably caused by differences in the scales’ assessment of ACh burden [3, 5, 10–14]. Compared to international scales, both German indicators (GerDBI and GerABS) indicated higher use of ACh medications in our study population. Despite the observed variation in prevalence, no differences were seen in the association between the various ACh burden measures and falls.

Studies comparing more than two measures of ACh burden and their relationship to clinical outcomes are rare. A recent systematic review by Lisibach et al. [5] identified only two such studies, one of which included falls as an outcome of interest [12]. Of nine ACh burden measures, four were significantly linked to falls, whereby the strongest association was found for the DBI, followed by the ARS, and the scales of Chew et al. and Sittironnarit et al. [61, 62]. While the significant association between the DBI and falls, and especially the advantage it offers of considering dose adjustments, has been described by other researchers [11, 15, 63], dose-dependent ACh measures included in our study (GerDBI and MARANTE) did not predict falls more accurately than any other scales. However, this conclusion is drawn based on the results of the complete-case analysis and should therefore be interpreted with caution as missing data on dosage have prevented us from calculating dose-dependent ACh burden for all participating patients. It should be further noted that the GerDBI includes both medication with anticholinergic and sedative activities.

A systematic review by Stewart et al. [21] compared eight studies of the relationship between the ARS and the Anticholinergic Cognitive Burden Scale (ACBS) [64] with falls. Consistent with our findings, the authors concluded that neither of the ACh measures could be explicitly identified as the best predictor of falls in older adults. Furthermore, results from a study by Ruxton et al. [65] indicate that the intake of individual medications such as imipramine or amitriptyline is more strongly associated with an increased risk of falls than ACh burden, as measured using scales. It is worth noting that the results of the studies described here showed great differences depending on the setting (e.g., primary care, nursing home, insurance database), follow-up duration (3–38.5 months), and the employed definition of falls (e.g., self-reported, falls reported in medical records) [12, 21, 65]. The limited comparability of our findings with previous research should therefore be taken into account when interpreting our results.

From a practical point of view, the results of our study call into question the use of ACh scales / equations to predict falls. On the other hand, although small, the added value of using symptoms to predict falls was nevertheless noteworthy. Attention should therefore be paid to the presence of adverse effects in patients taking an ACh medication, as they may indicate whether a patient is particularly sensitive. While the number of symptoms only showed a significant association in bivariate analyses, the association with dizziness / vertigo was statistically significant and present in all multivariate models. The strong association between previous falls and dizziness / vertigo and future falls has been identified in numerous other studies [66–70]. Dizziness / vertigo and balance problems have previously been linked to ACh burden, and can be clinically relevant when they increase the risk of falling [20]. Symptoms may therefore help to operationalize patients’ susceptibility and may be of interest in future research in this context. Further investigations should also consider the role of symptoms in relation to the intake of specific ACh medications and such fall-related outcomes as physical decline and fractures [71]. This is also true of other drugs that raise the risk of falls [72–74], even though most of them have been characterized to be of ACh nature [75]. An individual evaluation of patients’ medications (e.g., through a structured medication review [76]) in conjunction with a previous history of falls and dizziness / vertigo (as the strongest predictors identified in our study) should therefore be further explored.

To the best of our knowledge, this is the first study to explicitly investigate whether an individual’s risk of falling can be better predicted when ACh symptoms are considered in addition to qualitative and quantitative measures of ACh burden. With few exceptions, most studies examining the link between ACh measures and falls were conducted outside Germany [3, 5, 77] and used scales that had not been adapted for use in the German drug market. In our study, differences in the performance of the various tools were small, but the metric which performed best was one of the two German tools.

The likelihood of falls in older persons is dependent on an almost infinite number of factors. Risk and protective factors depend on a complex interplay between individual characteristics, such as age, morbidity, functional status, medication and behavioral patterns, and social and living environments. This poses limits to any attempt to explore causal and / or prognostic associations. The unavailability of data prevented us from considering known risk factors for falls in older patients such as gait problems or muscle weakness [67, 70], or from drawing conclusions for specific patient populations, such as people living with HIV [78]. It is also unclear whether a longer follow-up period or a different way of operationalizing falls (continuous count) would have resulted in different findings. Although the follow-up duration of 6 months was shorter than in most other studies [79, 80], another limitation was nonetheless the risk of recall bias when patients self-reported falls [81]. Furthermore, information on falls used in other analyses was mainly based on claims data and medical records, in which severe falls with serious consequences are preferentially recorded.

From a methodological point of view, the best-performing model yielded a *c*-statistic of 0.732, which indicates that the model had acceptable discrimination. External validation resulted in a decrease in the model’s discriminative ability, and calibration measures indicated that, to some degree, the model over-predicted and over-estimated the risk of falls. Sensitivity analysis indicated that intervention status had an impact on the model’s outcome. A general challenge to external model validation in different study cohorts has already been identified in the prediction of hospital admissions in PROPERmed [32]. In our case, this may be partly explained by differences in the inclusion criteria and in the baseline risk of patients from the development and validation datasets. On average, RIME patients were older (77 vs. 72 years), more frequently hospitalized, had more chronic conditions, took more drugs, and reported more symptoms than PRIMUM patients [30]. Other potential limitations relate to the harmonization of predictors, the insufficient sample sizes of the development and validation cohorts, and the low numbers of events (*n* = 188 in the development cohort and *n* = 55 in the validation cohort). As a rule-of-thumb, a minimum number of 100 events has been recommended for the external validation of a prognostic model [82]. It is also worth noting that our results reflect the data, ACh burden measures, and outcomes, selected for this study. It should therefore be borne in mind that our findings may have been different if we had chosen different measures of ACh burden or outcomes other than falls.

In conclusion, the findings of this study call into question the added value of using measures of ACh burden for risk classification in fall-prediction models. On the other hand, our findings indicate that the inclusion of symptom variables improve model performance. Medications that pose a risk of falling are a risk factor that can be directly modified, for example by changing the medication, or adjusting the dose. The prescription of medications with ACh properties in older patients should therefore be carefully evaluated, especially in patients with a history of falls and existing symptoms of dizziness / vertigo.

## Supporting information

S1 Text Adaptions to the study protocol. (PDF)

S1 Table Patient characteristics and results from the bivariate analysis of the complete-case population of the development set (n = 1,015). Abbreviations: ARS–Anticholinergic Risk Scale (23); ADS–Anticholinergic Drug Scale (41); GerABS–German Anticholinergic Burden Score (42); MARANTE–Muscarinic Acetylcholinergic Receptor ANTagonist Exposure Scale (18); GerDBI–German Drug Burden Index.
(DOCX)

S2 Table Base model for falls within 6-months of follow-up (Model 1). (DOCX)

S3 Table Comparison of model for falls within 6-months of follow-up from step 2. Abbreviations: ACh–anticholinergic; ARS–Anticholinergic Risk Scale (23); ADS–Anticholinergic Drug Scale (41); AIC–Akaike Information Criterion; GerABS–German Anticholinergic Burden Score (42); MARANTE–Muscarinic Acetylcholinergic Receptor ANTagonist Exposure Scale (18); GerDBI–German Drug Burden Index.
(PDF)

S4 Table Pooled estimates after multiple imputation. Abbreviation: GerABS–German Anticholinergic Burden Score (42).
(DOCX)

S5 Table Base model incl. symptoms for falls within 6-months of follow-up (Model 4). (DOCX)

S6 Table Sensitivity analysis. Abbreviation: CI - confidence interval.
(PDF)
