# Supplementary material for: Anticholinergic burden measures, symptoms, and fall-associated risk in older adults with polypharmacy: Development and validation of a prognostic model
Source: PLoS One. 2023 Jan 23;18(1):e0280907. doi: 10.1371/journal.pone.0280907 (PMC9870119; doi:10.1371/journal.pone.0280907)
Supplement: S1 Text — (PDF) [file pone.0280907.s001.pdf]

S1 Text. Adaptions to the study protocol

In the development of the baseline model in step 1, we originally planned to use sociodemographics / lifestyle, morbidity, and health-status / well-being as candidate predictors. In the next step, the number of drugs and ACh variables were then to be added to the base model. Instead, the number of drugs was already considered in step 1, so in step 2, only the added predictive value of using ACh variables could be quantified (step 2). After step 2, it was pre-specified to select the best-performing step 2 model for step 3 and internal validation. Rather than one model, all 15 models from step 2 were extended in step 3, and the best-performing model from step 3 was selected for internal validation. Following the protocol, the differences in discrimination between models were assessed by calculating differences in AUC. In addition, the increase in discrimination was quantified using IDI. In an additional exploratory analysis, a prognostic model was built (Model 4: base model + symptoms).
