# Supplementary material for: Anticholinergic burden measures, symptoms, and fall-associated risk in older adults with polypharmacy: Development and validation of a prognostic model
Source: PLoS One. 2023 Jan 23;18(1):e0280907. doi: 10.1371/journal.pone.0280907 (PMC9870119; doi:10.1371/journal.pone.0280907)
Supplement: S1 Table — Abbreviations: ARS–Anticholinergic Risk Scale (23); ADS–Anticholinergic Drug Scale (41); GerABS–German Anticholinergic Burden Score (42); MARANTE–Muscarinic Acetylcholinergic Receptor ANTagonist Exposure Scale (18); GerDBI–German Drug Burden Index. (DOCX) [file pone.0280907.s002.docx]

**S1 Table. Patient characteristics and results from the bivariate analysis of the complete-case population of the development set (n=1,015)**

| **Type** | **Variables** | **Categories / unit** | **Mean (SD) / median (IQR) /**  **number (%)** | **Missing values (%)** | **Odds Ratio**  **(95% CI)** | ***p*-value** |
| --- | --- | --- | --- | --- | --- | --- |
| Outcome | Falls | Yes | 188 (18.5%) | 9.5% | - | - |
| Socio-demographic and lifestyle-related | Intervention status | Intervention | 510 (50.2%) | 0% | 1.4 (1.0-1.9) | 0.04 |
|  | Age | Years | 76.7 (4.8) /  76 (7) |  | 1.0 (1.0-1.1) | 0.23 |
|  | Sex | Female | 507 (50.0%) | 0% | 1.6 (1.2-2.2) | 0.004 |
|  | Living situation | Living at home  Institutionalized living | 977 (96.3%)  38 (3.7%) | 0% | 1.4 (0.6-2.9) | 0.41 |
|  | Educational level | Low  Medium  High | 183 (18.0%)  747 (73.6%)  85 (8.4%) | 0% | 0.9 (0.6-1.4)  0.7 (0.3-1.3) | 0.77  0.27 |
|  | Smoking | Smoker  Ex-Smoker  Non-Smoker | 62 (6.1%)  486 (47.9%)  467 (46.0%) | 0% | 2.6 (1.1-7.5)  2.9 (1.2-8.4) | 0.05  0.03 |
| Morbidity-related | Hypertension | Yes | 899 (88.6%) | 0% | 1.6 (0.9-2.9) | 0.10 |
|  | Diabetes mellitus | Yes | 454 (44.7%) | 0% | 1.2 (0.9-1.7) | 0.19 |
|  | Coronary heart disease | Yes | 435 (42.9%) | 0% | 1.0 (0.8-1.4) | 0.82 |
|  | Osteoarthritis | Yes | 457 (45.0%) | 0% | 1.4 (1.0-2.0) | 0.03 |
|  | COPD/asthma | Yes | 226 (22.3%) | 0% | 0.8 (0.5-1.2) | 0.26 |
|  | Vision problems | Yes | 475 (46.8%) | 0% | 1.4 (1.0-1.9) | 0.05 |
|  | Hearing problems | Yes | 403 (39.7%) | 0% | 1.4 (1.0-2.0) | 0.03 |
|  | Cancer | Yes | 200 (19.7%) | 0% | 1.5 (1.0-2.1) | 0.04 |
|  | Heart failure | Yes | 334 (32.9%) | 0% | 0.9 (0.7-1.3) | 0.62 |
|  | Cerebrovascular disease | Yes | 137 (13.5%) | 0% | 1.2 (0.7-1.8) | 0.54 |
|  | Osteoporosis | Yes | 212 (20.9%) | 0% | 1.4 (1.0-2.0) | 0.08 |
|  | Depression | Yes | 121 (11.9%) | 0% | 1.5 (0.9-2.3) | 0.10 |
|  | Rheumatoid / seropositive arthritis | Yes | 196 (19.3%) | 0% | 1.1 (0.7-1.6) | 0.72 |
|  | Atherosclerosis / peripheral vascular disease | Yes | 308 (30.3%) | 0% | 1.4 (1.0-1.9) | 0.08 |
|  | Parkinsonism | Yes | 21 (2.1%) | 0% | 1.0 (0.3-2.8) | 0.95 |
|  | HIV / AIDS | Yes | 0 (0%) | 0% | - | - |
|  | Lipid disorder | Yes | 593 (58.4%) | 0% | 1.0 (0.7-1.3) | 0.76 |
|  | Gout | Yes | 304 (30.0%) | 0% | 1.0 (0.7-1.4) | 0.82 |
|  | Thyroid disorders | Yes | 318 (31.3%) | 0% | 1.4 (1.0-2.0) | 0.04 |
|  | Gastric or duodenal ulcer | Yes | 119 (11.7%) | 0% | 1.3 (0.8-2.0) | 0.32 |
|  | Liver disorder | Yes | 60 (5.9%) | 0% | 1.1 (0.6-2.1) | 0.76 |
|  | Urinary disease | Yes | 195 (19.2%) | 0% | 1.3 (0.9-1.9) | 0.16 |
|  | Anemia | Yes | 103 (10.2%) | 0% | 1.8 (1.1-2.8) | 0.02 |
|  | No. of chronic conditions | Frequency | 6.5 (2.5) /  6 (3) | 0% | 1.1 (1.1-1.2) | <0.001 |
| Health-status and well-being related | Pain | Yes | 742 (73.1%) | 0.9% | 2.8 (1.8-4.6) | <0.001 |
|  | Quality of life | Score | 0.7 (0.2) /  0.7 (0.2) | 3.1% | 0.1 (0.1-0.2) | <0.001 |
|  | Functional status | Score | 2.7 (2.5) /  2 (3) | 1.0% | 1.2 (1.1-1.3) | <0.001 |
|  | Cognitive impairment | Severe  Medium  Mild  No | 442 (43.5%)  232 (22.9%)  150 (14.8%)  191 (18.8%) | 1.2% | 1.06 (0.7-1.6)  0.8 (0.5-1.3)  1.3 (0.8-1.9) | 0.78  0.34  0.26 |
|  | All-cause hospital admissions | Yes | 376 (37.0%) | 0.3% | 0.6 (0.4-0.9) | 0.01 |
|  | History of falls at baseline | 0-1 fall  ≥ 2 falls | 965 (95.1%)  50 (4.9%) | 0% | 5.9 (3.3-10.6) | <0.001 |
| Medication-related | No. of drugs | Frequency | 8.5 (2.6) /  8 (3) | 0% | 1.1 (1.1-1.2) | <0.001 |
|  | ARS binary | Yes | 125 (12.3%) | 0% | 1.3 (0.8-2.0) | 0.34 |
|  | ARS count | Frequency | 0.1 (0.4) /  0 (0) | 0% | 1.3 (0.9-1.8) | 0.23 |
|  | ARS burden | Score | 0.2 (0.8) /  0 (0) | 0% | 1.2 (1.0-1.4) | 0.11 |
|  | ADS binary | Yes | 385 (37.9%) | 0% | 1.2 (0.9-1.7) | 0.27 |
|  | ADS count | Frequency | 0.5 (0.7) /  0 (1) | 0% | 1.3 (1.0-1.6) | 0.02 |
|  | ADS burden | Score | 0.7 (1.1) /  0 (1) | 0% | 1.2 (1.1-1.4) | <0.001 |
|  | GerABS binary | Yes | 792 (78.0%) | 0% | 1.1 (0.7-1.6) | 0.80 |
|  | GerABS count | Frequency | 1.3 (1.1) /  1 (1) | 0% | 1.2 (1.0-1.3) | 0.03 |
|  | GerABS burden | Score | 1.6 (1.5) /  1 (1) | 0% | 1.2 (1.1-1.3) | <0.001 |
|  | MARANTE binary | Yes | 201 (19.8%) | 0% | 1.5 (1.0-2.2) | 0.03 |
|  | MARANTE count | Frequency | 0.2 (0.5) /  0 (0) | 0% | 1.4 (1.1-1.8) | 0.02 |
|  | MARANTE burden | Score | 0.4 (1.0) /  0 (0) | 0% | 1.2 (1.1-1.4) | <0.001 |
|  | GerDBI binary | Yes | 531 (52.3%) | 0% | 1.1 (0.8-1.5) | 0.66 |
|  | GerDBI count | Frequency | 0.9 (1.1) /  0 (1) | 0% | 1.2 (1.0-1.3) | 0.02 |
|  | GerDBI burden | Score | 0.5 (0.7) /  0 (0.8) | 0% | 1.3 (1.0-1.6) | 0.04 |
| Symptoms | No. of symptoms | Frequency | 2.2 (1.6) /  2 (2) | 0% | 1.23 (1.11-1.36) | <0.001 |
|  | Dizziness / vertigo | Yes | 414 (40.8%) | 0.3% | 2.3 (1.7-3.2) | <0.001 |
|  | Problems urinating | Yes | 192 (18.9%) | 0.1% | 1.2 (0.8-1.8) | 0.36 |
|  | Stomach pain | Yes | 172 (16.9%) | 0.2% | 0.9 (0.6-1.4) | 0.69 |
|  | Drowsiness / fatigue | Yes | 419 (41.3%) | 0.4% | 1.4 (1.0-2.0) | 0.03 |
|  | Dry mouth | Yes | 475 (46.8%) | 0.4% | 1.3 (1.0-1.8) | 0.07 |
|  | Itching | Yes | 280 (27.6%) | 0.3% | 1.4 (1.0-1.9) | 0.07 |
|  | Constipation | Yes | 289 (28.5%) | 0.2% | 1.5 (1.0-2.0) | 0.03 |

Abbreviations: ARS – Anticholinergic Risk Scale (23); ADS – Anticholinergic Drug Scale (41); GerABS – German Anticholinergic Burden Score (42); MARANTE – Muscarinic Acetylcholinergic Receptor ANTagonist Exposure Scale (18); GerDBI – German Drug Burden Index.
