# Supplementary material for: Anticholinergic burden measures, symptoms, and fall-associated risk in older adults with polypharmacy: Development and validation of a prognostic model
Source: PLoS One. 2023 Jan 23;18(1):e0280907. doi: 10.1371/journal.pone.0280907 (PMC9870119; doi:10.1371/journal.pone.0280907)
Supplement: S2 Table — (DOCX) [file pone.0280907.s003.docx]

**S2 Table. Base model for falls within 6-months of follow-up (Model 1)**

| **Intercept and predictors** | **Unit** | **Regression Coefficient** | **Standard Error** | ***P*-value** |
| --- | --- | --- | --- | --- |
| Intercept |  | -3.36 | 1.49 | 0.02 |
| History of falls at baseline | ≥ 2 falls | 1.59 | 0.32 | <0.001 |
| Pain | Yes | 0.70 | 0.24 | 0.002 |
| All-cause hospital admissions | Yes | -0.49 | 0.19 | 0.02 |
| COPD / asthma | Yes | -0.57 | 0.22 | 0.02 |
| Functional status | Score | 1.06 | 0.35 | <0.001 |
| Hearing problems | Yes | 0.30 | 0.17 | 0.08 |
| Intervention status | Intervention | 0.29 | 0.17 | 0.08 |
| Sex | Female | 0.35 | 0.19 | 0.01 |
| Age | Years | -0.19 | 0.19 | 0.30 |
| No. of drugs | Frequency | 0.86 | 1.88 | 0.80 |
