# Supplementary material for: Anticholinergic burden measures, symptoms, and fall-associated risk in older adults with polypharmacy: Development and validation of a prognostic model
Source: PLoS One. 2023 Jan 23;18(1):e0280907. doi: 10.1371/journal.pone.0280907 (PMC9870119; doi:10.1371/journal.pone.0280907)
Supplement: S3 Table — Abbreviations: ACh–anticholinergic; ARS–Anticholinergic Risk Scale (23); ADS–Anticholinergic Drug Scale (41); AIC–Akaike Information Criterion; GerABS–German Anticholinergic Burden Score (42); MARANTE–Muscarinic Acetylcholinergic Receptor ANTagonist Exposure Scale (18); GerDBI–German Drug Burden Index. (PDF) [file pone.0280907.s004.pdf]

S3 Table. Comparison of model for falls within 6-months of follow-up from step 2

Model	Predictors	*c*-statistic	AIC
2.1	Base model + ARS binary	0.712	900.51
2.2	Base model + ARS count	0.712	900.60
2.3	Base model + ARS burden	0.712	900.88
2.4	Base model + ADS binary	0.714	898.98
2.5	Base model + ADS count	0.712	900.61
2.6	Base model + ADS burden	0.714	898.98
2.7	Base model + MARANTE binary	0.712	900.88
2.8	Base model + MARANTE count	0.712	900.88
2.9	Base model + MARANTE burden	0.712	900.71
2.10	Base model + GerABS binary	0.713	900.35
2.11	Base model + GerABS count	0.712	900.88
2.12	Base model + GerABS burden	0.711	900.46
2.13	Base model + GerDBI binary	0.712	899.37
2.14	Base model + GerDBI count	0.714	900.15
2.15	Base model + GerDBI burden	0.713	899.89

Abbreviations: ACh – anticholinergic; ARS – Anticholinergic Risk Scale (23); ADS – Anticholinergic Drug Scale (41); AIC – Akaike Information Criterion; GerABS – German Anticholinergic Burden Score (42); MARANTE – Muscarinic Acetylcholinergic Receptor ANTagonist Exposure Scale (18); GerDBI – German Drug Burden Index.
