# Supplementary material for: Anticholinergic burden measures, symptoms, and fall-associated risk in older adults with polypharmacy: Development and validation of a prognostic model
Source: PLoS One. 2023 Jan 23;18(1):e0280907. doi: 10.1371/journal.pone.0280907 (PMC9870119; doi:10.1371/journal.pone.0280907)
Supplement: S4 Table — Abbreviation: GerABS–German Anticholinergic Burden Score (42). (DOCX) [file pone.0280907.s005.docx]

**S4 Table. Pooled estimates after multiple imputation**

| **Intercept and predictors** | **Unit** | **Regression Coefficients** | **Standard Error** | ***P*-value** |
| --- | --- | --- | --- | --- |
| Intercept |  | -3.40 | 1.37 | 0.01 |
| History of falls at baseline | ≥ 2 falls | 1.61 | 0.31 | <0.001 |
| Dizziness / vertigo | Yes | 0.54 | 0.17 | 0.002 |
| COPD / asthma | Yes | -0.54 | 0.21 | 0.01 |
| Pain | Yes | 0.61 | 0.24 | 0.01 |
| All-cause hospital admissions | Yes | -0.44 | 0.18 | 0.01 |
| Functional status | Score | 0.86 | 0.36 | 0.02 |
| Stomach pain | Yes | -0.48 | 0.26 | 0.04 |
| Intervention status | Intervention | 0.22 | 0.18 | 0.23 |
| Hearing problems | Yes | 0.23 | 0.16 | 0.17 |
| Cancer | Yes | 0.36 | 0.19 | 0.07 |
| No. of drugs | Frequency | 0.79 | 0.30 | 0.01 |
| GerABS binary | Yes | -0.11 | 0.21 | 0.60 |
| Sex | Female | 0.35 | 0.17 | 0.04 |
| Age | Years | 0.03 | 1.73 | 0.99 |

Abbreviation: GerABS – German Anticholinergic Burden Score (42).
