# Supplementary material for: Anticholinergic burden measures, symptoms, and fall-associated risk in older adults with polypharmacy: Development and validation of a prognostic model
Source: PLoS One. 2023 Jan 23;18(1):e0280907. doi: 10.1371/journal.pone.0280907 (PMC9870119; doi:10.1371/journal.pone.0280907)
Supplement: S5 Table — (DOCX) [file pone.0280907.s006.docx]

**S5 Table. Base model incl. symptoms for falls within 6-months of follow-up (Model 4)**

| **Intercept and predictors** | **Unit** | **Regression Coefficients** | **Standard Error** | ***P*-value** |
| --- | --- | --- | --- | --- |
| Intercept |  | -3.60 | 1.51 | 0.02 |
| History of falls at baseline | ≥ 2 falls | 1.53 | 0.32 | <0.001 |
| Dizziness / vertigo | Yes | 0.60 | 0.18 | 0.001 |
| COPD / asthma | Yes | -0.58 | 0.22 | 0.01 |
| Pain | Yes | 0.67 | 0.25 | 0.006 |
| All-cause hospital admissions | Yes | -0.49 | 0.19 | 0.009 |
| Functional status | Score | 0.92 | 0.37 | 0.02 |
| Stomach pain | Yes | -0.49 | 0.24 | 0.04 |
| Intervention status | Intervention | 0.25 | 0.18 | 0.16 |
| Hearing problems | Yes | 0.26 | 0.18 | 0.14 |
| Cancer | Yes | 0.32 | 0.21 | 0.13 |
| No. of drugs | Frequency | 0.84 | 0.32 | 0.01 |
| Sex | Female | 0.35 | 0.19 | 0.05 |
| Age | Years | -0.06 | 1.91 | 0.98 |
