# Supplementary material for: Anticholinergic burden measures, symptoms, and fall-associated risk in older adults with polypharmacy: Development and validation of a prognostic model
Source: PLoS One. 2023 Jan 23;18(1):e0280907. doi: 10.1371/journal.pone.0280907 (PMC9870119; doi:10.1371/journal.pone.0280907)
Supplement: S6 Table — Abbreviation: CI - confidence interval. (PDF) [file pone.0280907.s007.pdf]

S6 Table. Sensitivity analysis

Model	c-statistic
Base model (incl. intervention status)	0.712 (CI: 0.672-0.752)
Base model (excl. intervention status)	0.710 (CI: 0.670-0.751)
Full model (incl. intervention status)	0.732 (CI: 0.694-0.769)
Full model (excl. intervention status)	0.727 (CI: 0.688-0.766)
Externally validated model (incl. intervention status)	0.632 (CI: 0.543-0.721)
Externally validated model (excl. intervention status)	0.635 (CI: 0.548-0.722)

CI – confidence interval.
